# 

**Published:** 1872-12

**Authors:** 


MEETING OF THE NEW YORK STATE MEDICAL SOCIETY.—The New York State Medical Society holds its annual meeting in Albany the first Tuesday in February, 1873. Delegates from County Medical Societies should not fail to be present. So few have attended from Erie county that there are not any physicians eligible to permanent membership at the present time, we believe.

### Books and Pamphlets Received.

The Microscope and Microscopical Technology. A text-book for physicians and students. By Henrich Frey, Professor of Medicine in Zurich, Switzerland. Translated from the German and edited by Geo. R. Cutter, M. D. New York: Wm. Wood & Co., 1872. Buffalo: H. H. Otis.

The Pathology, Diagnosis and Treatment of Diseases of Women, including the Diagnosis of Pregnancy. By Grailey Hewitt, M. D., London, F. R. C. P. Second American from the Third London Edition. Philadelphia: Lindsay & Blakiston, 1872; Buffalo: T. Butler & Son.

A System of Oral Surgery; being a Consideration of the Diseases and Surgery of the Mouth, Jaws, and Associate Parts. By James E. Garretson, M. D., D. D. S. Philadelphia: J. B. Lippincott & Co., 1872; Buffalo: H. H. Otis.

Questions in Surgery. By Wm. Warren Green, M. D.

Annual Report of the Surgeon-General United States Army.

Thirteenth Annual Report of the Superintendent of the State Lunatic Asylum for Insane Criminals, at Auburn, N. Y.

An Examination of Prof. Reese's Review of the Trial of Mrs. Wharton for the Murder of General Ketchum. By Philip C. Williams, M. D. Reprinted from *Medical and Surgical Reporter*.

The Use of the Seton in the Treatment of some Cases of Chronic Affections of the Womb. By Ely Van De Warker, M. D. Reprinted from the American Journal of Obstetrics.

Medical Responsibility and Malpractice. An Address delivered before the Medical Society of the State of New York at its Sixty-sixth Annual Meeting, February 7, 1872. By Wm. C. Wey, M. D., President of the Society.

Medico-Legal Society of the city of New York.

THE BUFFALO

### Medical and Surgical Journal

PUBLISHED MONTHLY,

Containing Original Articles, Reports of Medical Societies and Hospitals, Editorial, Reviews, Correspondence, Army News, etc., etc.; including the usual variety of Medical Periodical Publications. Specimen copies sent on application.

Address Buffalo Medical & Surgical Journal, Buffalo, N. Y.

TERMS OF SUBSCRIPTION, - - $3.00 PER YEAR, IN ADVANCE.

